# Pediatric Spontaneous Pneumomediastinum after a Push-Up Exercise: An Uncommon Complication of a Common Exercise

**DOI:** 10.3390/children7120287

**Published:** 2020-12-11

**Authors:** Chih-Yi Tsay, Yu-Long Chen, Chien-Sheng Chen, Po-Chen Lin, Meng-Yu Wu

**Affiliations:** 1 Department of Emergency Medicine, Taipei Tzu Chi Hospital, Buddhist Tzu Chi Medical Foundation, New Taipei City 231, Taiwan; belle99311101@gmail.com (C.-Y.T.); yulong0129@gmail.com (Y.-L.C.); holeyeye@yahoo.com.tw (C.-S.C.); taipeitzuchier@gmail.com (P.-C.L.); 2 Department of Emergency Medicine, School of Medicine, Tzu Chi University, Hualien 970, Taiwan

**Keywords:** pediatric, spontaneous pneumomediastinum, exercise-related, subcutaneous emphysema

## Abstract

Pediatric spontaneous pneumomediastinum is an uncommon condition associated with infection, trauma, or coexisting structural lung pathology. Exercise-related spontaneous subcutaneous emphysema and pneumomediastinum are rarely reported. However, severe pneumomediastinum may coexist with pneumothorax, pneumorrhachis, and subcutaneous emphysema, which can potentially lead to serious complications, including airway obstruction and pneumorrhachis. Therefore, early diagnosis and timely management are important for physicians to determine the etiology and prevent further damage. Here, we present a case of exercise-related spontaneous subcutaneous emphysema and pneumomediastinum to highlight the pathogenesis and suggest therapeutic strategies.

## 1. Introduction

Pediatric spontaneous pneumomediastinum is a rare presentation associated with infection, trauma, or coexisting structural lung pathology. Chalumeau et al. [1] analyzed its common clinical symptoms, including chest pain, dyspnea, cough, and neck pain. The pathogenesis of spontaneous pneumomediastinum is reported to involve high intra-alveolar and intrathoracic pressures that may cause the rupture of the small alveoli. Air leaks from the surrounding bronchovascular sheath spread to the upper respiratory tract, intrathoracic airways, or esophageal tract. Forceful coughing leads to air leaks from the site of rupture of the alveoli or bronchioles, causing subcutaneous emphysema [2]. Common triggers in the pediatric population are asthma, vomiting, and the Valsalva maneuver. The evaluation of etiology should include examinations regarding trauma history and underlying lung disease. Spontaneous pneumomediastinum is usually an isolated finding, with no significant complications. However, lung infections, such as tuberculosis, may lead to structural lung pathology and weakening of the alveoli or bronchioles, causing spontaneous pneumomediastinum. In very rare conditions, increased pressure in the mediastinal cavity may also cause pneumothorax, tension pneumomediastinum, tension pneumopericardium, pneumorrhachis, and subcutaneous emphysema, which may potentially cause serious complications, including airway obstruction and neurological defects [1,3,4,5,6]. Therefore, early diagnosis is important for emergency physicians to identify the etiology and prevent catastrophic complications. Here, we present a case of exercise-related spontaneous subcutaneous emphysema and pneumomediastinum.

## 2. Case Presentation

**Figure 1:**
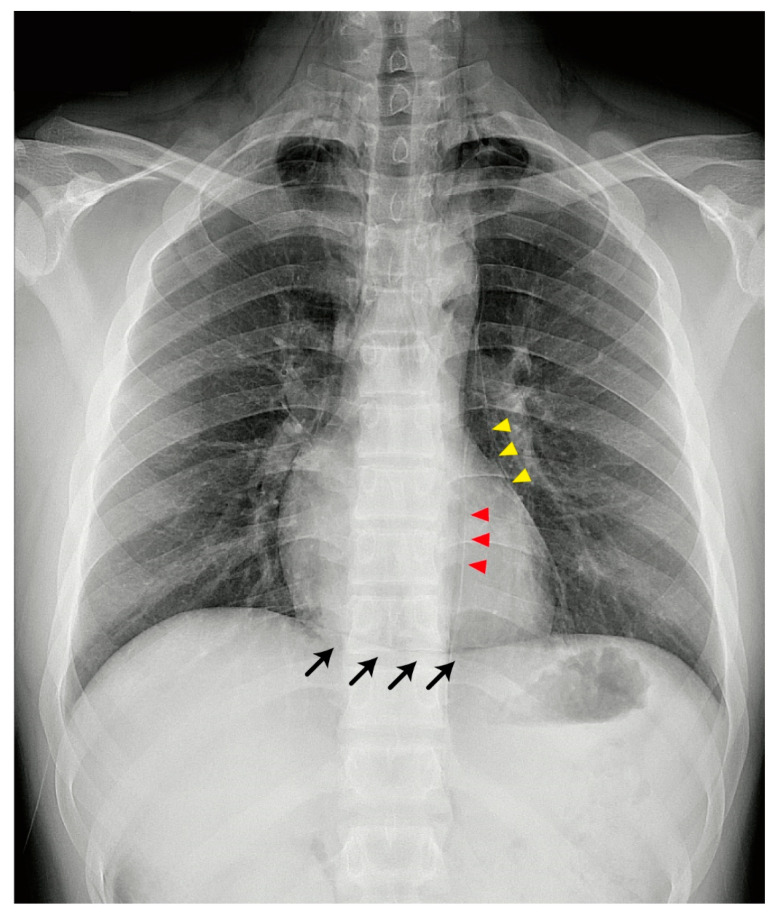
Chest X-ray shows gross subcutaneous emphysema over the neck and pneumomediastinum with a continuous diaphragm sign (black arrows). The gas outlining the mediastinum, aorta (red arrowheads), and heart (yellow arrowheads) extends into the neck.

**Figure 2:**
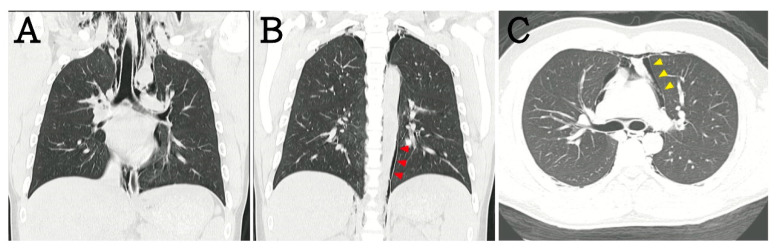
In computed tomography (CT) scans, these results were also noted, including (**A**) subcutaneous emphysema, (**B**) periaortic gas, and (**C**) pericardiac gas.

A 16-year-old boy without asthma or smoking history presented with the sudden onset of retrosternal chest pain for two days after performing a 4-h push-up exercise. The other symptoms included chest tightness, throat pain, and swelling over the neck. There was no dyspnea, cold sweating, or radiating pain. The primary examination revealed a blood pressure of 131/72 mmHg, heart rate of 79 beats/min, and oxygen saturation (SpO_2_) of 96%. On examination, a crepitus was palpated over the neck and chest wall. Cardiac auscultation showed the presence of Hamman’s sign. Electrocardiography revealed normal sinus rhythm without QTc prolongation or axis deviation. Laboratory analysis revealed that high-sensitivity troponin-I levels were within the normal range. Chest radiography showed gross subcutaneous emphysema over the neck and pneumomediastinum with a continuous diaphragm sign (black arrows; Figure 1). The gas outlining the mediastinum, aorta (red arrowheads), and heart (yellow arrowheads) extended into the neck. Computed tomography (CT) revealed cervical subcutaneous emphysema and pneumomediastinum dissecting into the retroperitoneum (Figure 2). After three days of observation and oxygenation, the subcutaneous emphysema and pneumomediastinum improved. The patient was regularly followed-up at the outpatient department. We have received a written informed consent form from the patient.

## 3. Discussion

Exercise-induced pneumomediastinum is a rare condition, especially in pediatric populations. The most common symptoms of spontaneous subcutaneous pneumomediastinum are retrosternal chest pain and dyspnea. Other symptoms include throat pain, neck pain, odynophagia, and dysphagia [7,8,9]. Subcutaneous emphysema is an important sign in pediatric patients with exercise-related pneumomediastinum. Exercise-related spontaneous pneumomediastinum is commonly found in high-school-aged populations. Mihos et al. [10] reported four forceful exercise activities, including scuba diving, basketball, soccer, and volleyball, related to spontaneous pneumomediastinum. A traumatic event during exercise may be a cause of pneumomediastinum. However, in our case, forceful breath-holding during exercise may have led to pneumomediastinum. The mechanism is similar to that in scuba diving, wherein high intra-alveolar pressure can cause rupture and air leak. For this reason, the physician must identify forceful traumatic or breath-holding events and check for subcutaneous emphysema. Once pneumomediastinum is suspected, chest radiography and CT are effective in detecting air leaks in the bronchovascular sheath (Figure 1) [11]. The prognosis of pneumomediastinum is based on its etiology. Complicated pneumomediastinum may be induced by pneumothorax or esophageal perforation, which requires intensive medical or surgical management. Early identification of complicated pneumomediastinum and the exclusion of acute cardiac events, such as myocarditis, myocardial infarction, cardiac tamponade, and traumatic cardiac injury, can prevent catastrophic complications. The treatment of uncomplicated pneumomediastinum is directed toward symptom relief. Although uncomplicated pneumomediastinum is self-limiting, patients need to be monitored for complications such as tension pneumomediastinum [3], pneumopericardium [4], and pneumorrhachis [5]. Tension pneumomediastinum is usually associated with mechanical ventilation intervention due to increasing massive pneumomediastinum [1]. Tension pneumopericardium is more commonly found in newborn infants receiving mechanical ventilation [12,13]. The incidence of these complications is rare, and they have only been reported in a few case reports, but physicians should keep these complications in mind, especially while treating patients receiving mechanical ventilation. 

## 4. Conclusions

In conclusion, spontaneous pneumomediastinum is a rare and benign disease with a good prognosis. Exercise-related spontaneous pneumomediastinum should be suspected in pediatric patients who develop subcutaneous emphysema after exercise.
